# Supplementary material for: Layer-selective hydrogenation and proton transport in twisted bilayer graphene
Source: Nat Commun. 2026 Apr 4;17:4873. doi: 10.1038/s41467-026-71147-y (PMC13230707; doi:10.1038/s41467-026-71147-y)
Supplement: Supplementary file 1 — Supplementary Information [file 41467_2026_71147_MOESM1_ESM.pdf]

Supplementary Information

Layer-selective hydrogenation and proton transport in twisted bilayer graphene

J. Tong[1,2*], G. Chen[1,2], H. Li[1], E. Hoenig[1,2], M. Alhashmi[1,2], X. Zhang[1,2], D. Bahamon[3,4], G. R. Tainton[2,5], S. Sullivan-Allsop[2,5], Y. Mayamei[1,2], D. R. da Costa[6], L. F. Vega[3,4], S. J. Haigh[2,5], D. Domaretskiy[1], F. M. Peeters[6,7,8], M. Lozada-Hidalgo[1,2*]

[1] Department of Physics and Astronomy, The University of Manchester, Manchester M13 9PL, UK
[2] National Graphene Institute, The University of Manchester, Manchester M13 9PL, UK
[3]Research and Innovation Center on CO2 and Hydrogen (RICH Center) and Chemical Engineering Department, Khalifa University, PO Box 127788, Abu Dhabi, United Arab Emirates
[4]Research and Innovation Center for graphene and 2D materials (RIC2D), Khalifa University, PO Box 127788, Abu Dhabi, United Arab Emirates
[5] Department of Materials, The University of Manchester, Manchester M13 9PL, UK
[6]Departamento de Física, Universidade Federal do Ceará, 60455-900 Fortaleza, Ceará, Brazil
[7]School of Physics and Optoelectronic Engineering, Nanjing University of Information Science and Technology, Nanjing 210044, China.
[8]Departement Fysica, Universiteit Antwerpen, Groenenborgerlaan 171, B-2020 Antwerp, Belgium
*tongjincheng@outlook.com; marcelo.lozadahidalgo@manchester.ac.uk

The PDF file includes:

Supplementary Figures

Supplementary Fig. 1 | Twisted bilayer graphene device fabrication.
Supplementary Fig. 2 | Raman measurement of twisted bilayer graphene devices.
Supplementary Fig. 3 | Independence of top and bottom gates.
Supplementary Fig. 4 | DFT calculations of proton transport and hydrogenation in twisted bilayer graphene.
Supplementary Fig. 5 | In situ Raman spectra during electrochemical hydrogenation of twisted bilayer graphene.
Supplementary Fig. 6 | Selective hydrogenation cyclability.
Supplementary Fig. 7 | Natural bilayer graphene.
Supplementary Fig. 8 | Electrostatic model of twisted bilayer graphene.
Supplementary Fig. 9 | Conductance map of twist bilayer graphene shows the splitting of the neutrality lines.

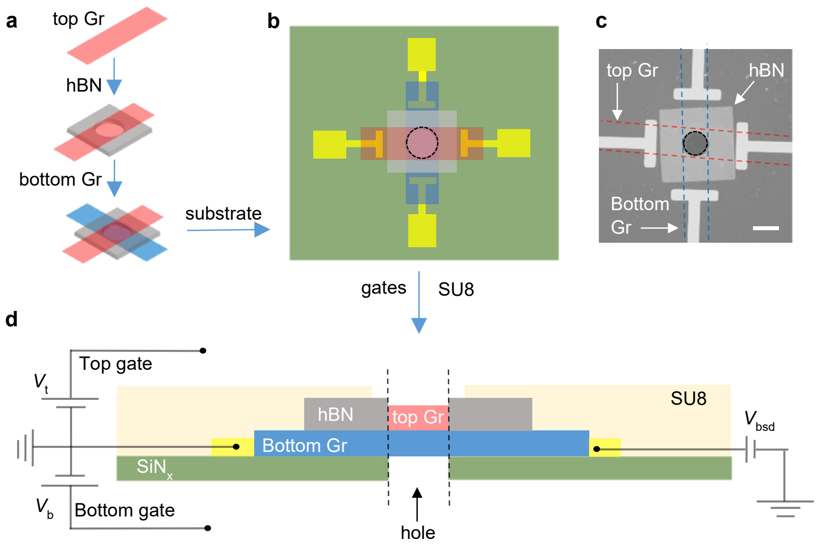

Supplementary Fig. 1 | Twisted bilayer graphene device fabrication. a, Two graphene monolayers are stacked on either side of a thick hBN flake with a circular hole in the middle. This configuration ensures that the two flakes are not electrically connected outside the circular hole. **b**, The graphene/hBN/graphene stack is transferred onto a SiN_x substrate containing a circular aperture aligned with the aperture in the hBN flake. **c**, Optical image of the device after the transfer of graphene/hBN/graphene stack. Scale bar, 10 µm. **d**, Schematic of the electrical circuit and the cross section of the finished device after the SU8 clamp was transferred on top to isolate the contacts from the electrolyte.

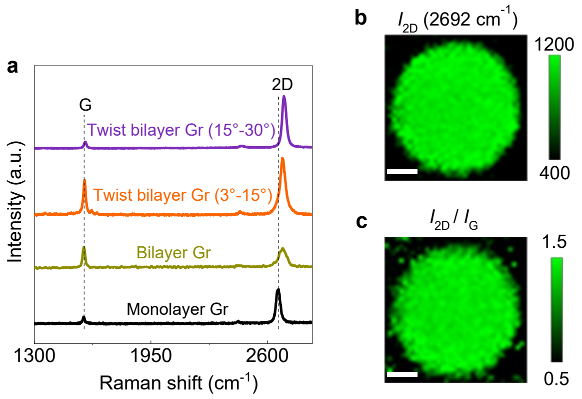

Supplementary Fig. 2 | Raman measurement of twisted bilayer graphene devices. a, Raman spectra of monolayer (black), natural bilayer (gold), and two typical twisted bilayer graphene devices (orange and purple). Dashed lines indicate the positions of the G, and 2D Raman bands. **b**, Spatial map of the G band of a typical twisted bilayer suspended device. **c**, Spatial map of the I_{2D}/I_G ratio of a typical twisted bilayer suspended device. Scale bars, 2.5 µm.

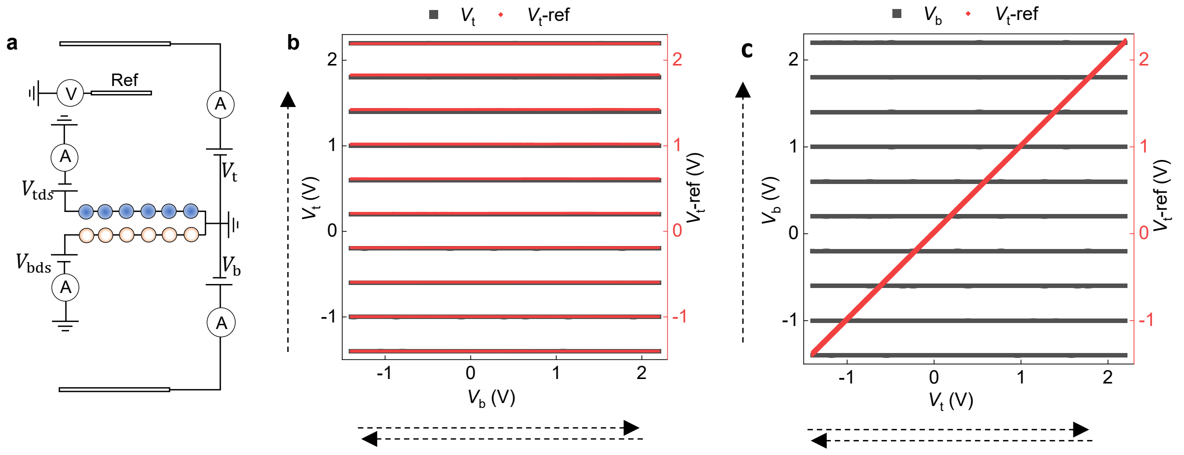

Supplementary Fig. 3 | Independence of top and bottom gates. a, Schematic of experimental setup. **b,** Reference electrode voltage, V_t^{ref} (red), measured as a function of bottom gate voltage V_b, while the top gate voltage V_t (black) is held fixed. The bottom gate is swept repeatedly at each fixed V_t (horizontal dashed arrows). The vertical dashed line marks steps in V_t. Left y-axis (black): applied V_t. Right *y*-axis (red): measured V_t^{ref}. **c,** V_t^{ref} (red) as a function of V_t for fixed V_b (black). Left *y*-axis (black): applied V_b. Right y-axis (red): measured V_t^{ref}. Horizontal and vertical lines indicate the respective sweeping and stepping of gate voltages.

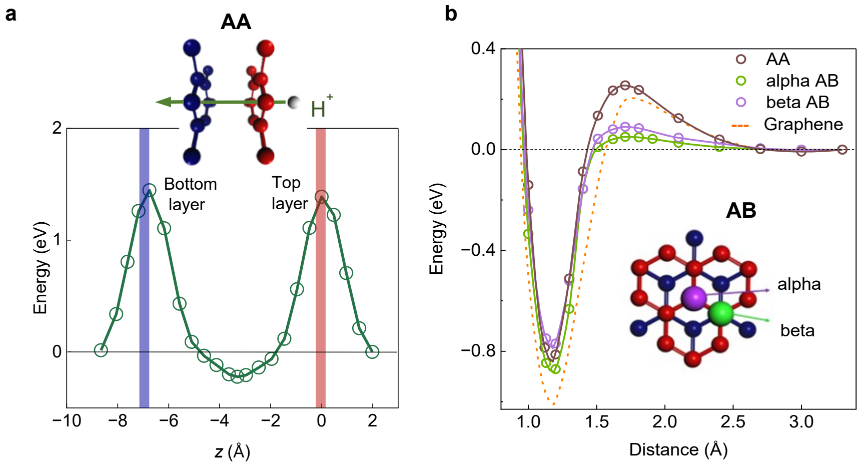

Supplementary Fig. 4 | DFT calculations of proton transport and hydrogenation in twisted bilayer graphene. a, Top: schematic of proton permeation through the AA region of twisted bilayer graphene. White sphere: proton; red and blue spheres: carbon atoms in the top and bottom layers, respectively. Main panel: potential energy profile along the proton's path perpendicular to the basal plane through the AA-stacked region. Red and blue bands mark the positions of the top and bottom graphene layers. **b,** Potential energy profile of a proton above a carbon atom in twisted bilayer graphene for different stack configurations (AA, and two sites in the AB configuration). Inset: schematic of AB-stacked hydrogenation sites on the top layer (red). In the α site, a carbon sits over the centre of the hexagonal ring in the previous layer, whereas in the β site it sits over a carbon atom. The energy profile for a proton above a carbon atom in monolayer graphene (orange) is shown for reference.

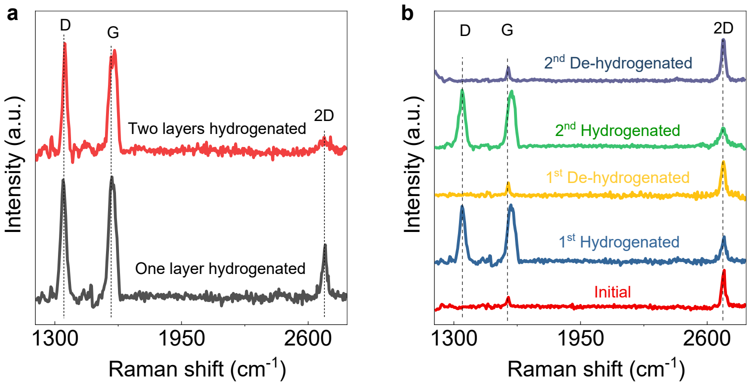

Supplementary Fig. 5 | In situ Raman spectra during electrochemical hydrogenation of twisted bilayer graphene. a, Raman spectra obtained when only one of the layers (black) and when both layers are hydrogenated (red). **b**, The devices can undergo multiple cycles of single-layer hydrogenation and dehydrogenation without degradation. Dashed lines indicate the positions of the D, G, and 2D Raman bands.

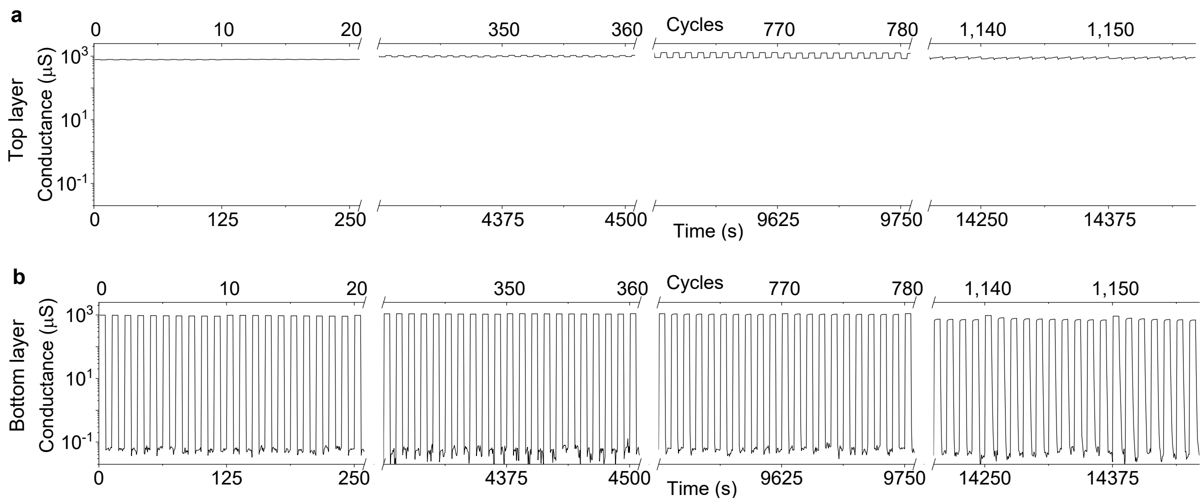

Supplementary Fig. 6 | Selective hydrogenation cyclability. Conductance of **a,** top layer and **b,** bottom layer graphene as a function of cycles over time. For each cycle (12.5 s), gate voltages of V_t and V_b were set to 0.9 V and 1.9 V, respectively, to hydrogenate the top layer while to keep the bottom layer remain conductive. For dehydrogenation of the top layer, both V_t and V_b were set to be -1 V.

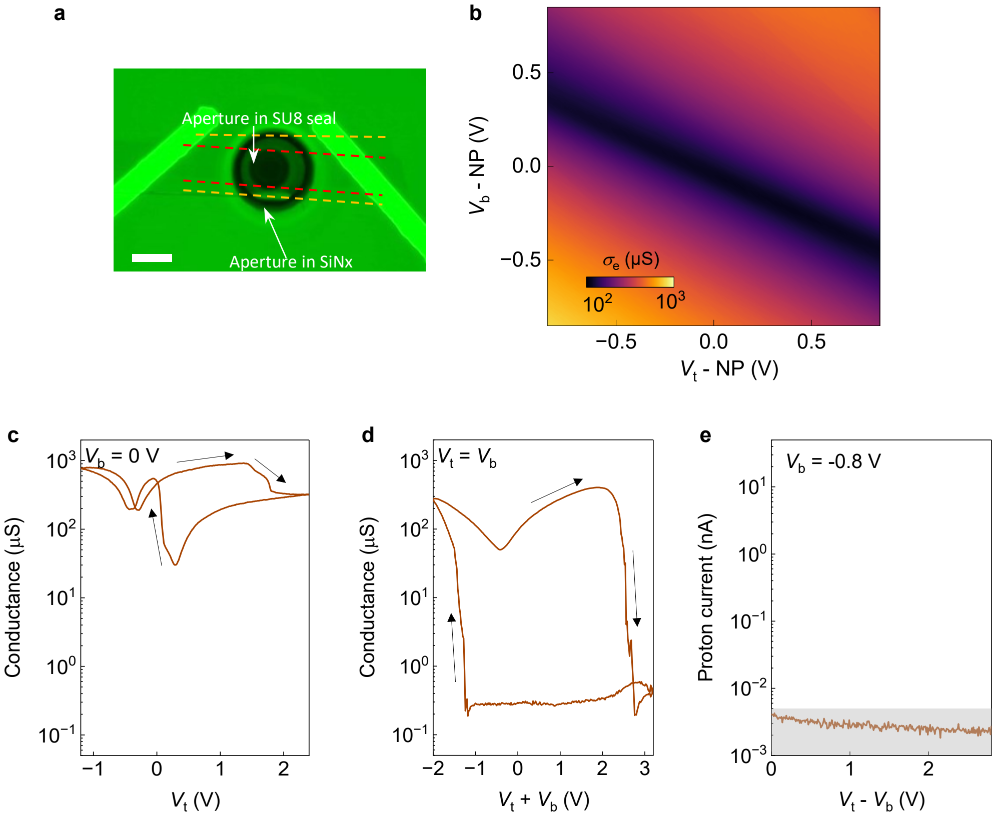

Supplementary Fig. 7 | Natural bilayer graphene. a, Optical image of AB stacked bilayer graphene device. Dashed lines mark the boundaries of each layer in the flake. Scale bar, 10 μm. **b**, Map of the in-plane electronic conductance (σ_e) as a function of V_t and V_b vs NP. Only one neutrality line is observed. **c**, In-plane electronic conductance of natural bilayer graphene as a function of V_t along the loop marked with red arrows with V_b is set to 0. There is a clear conductance drop after V_t of 1.5 V, revealing the hydrogenation of the top layer, while the bilayer remains conductive because the bottom layer is still metallic. **d**, In-plane electronic conductance of natural bilayer graphene as a function of $V_t + V_b$ along the loop marked with red arrows with $V_t = V_b$. A full conductor to insulator transition was observed at $V_t + V_b = 2.8$ V as both layers are hydrogenated. **e**, Proton current as a function of the electric field (V_t - V_b) at constant V_b of -0.8 V, confirming no proton transport over AB stacked bilayer graphene. Grey area, resolution background determined by parasitic leakage currents.

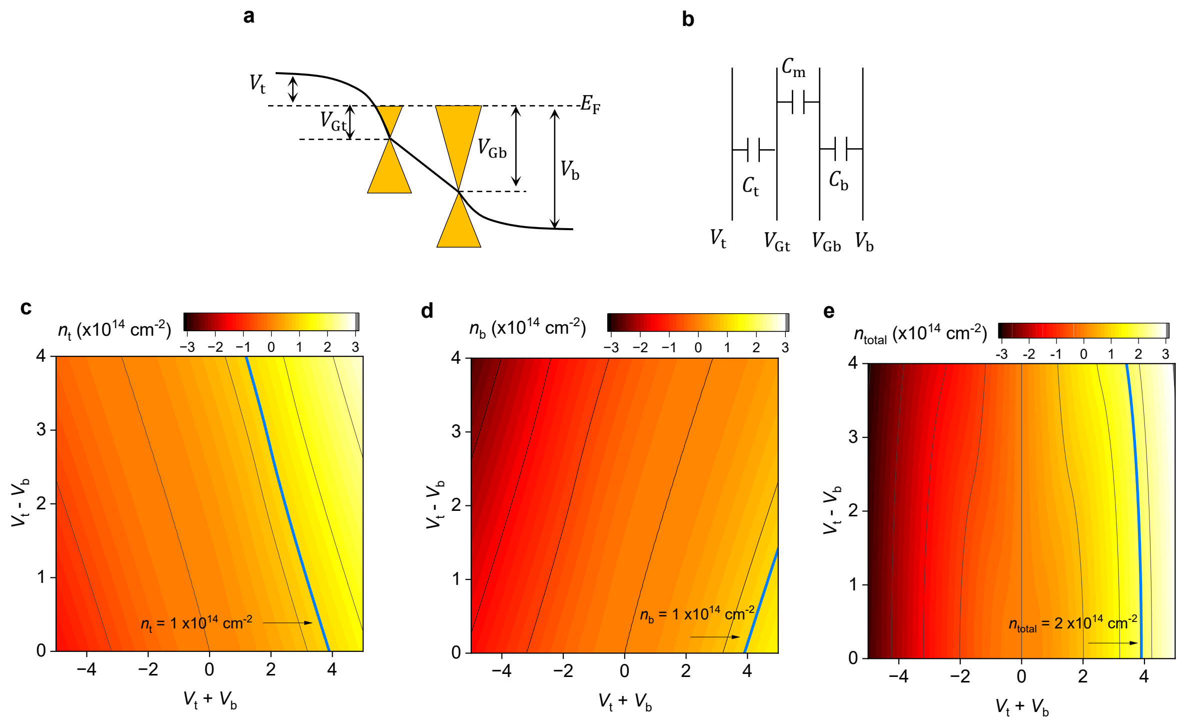

Supplementary Fig. 8 | Electrostatic model of twisted bilayer graphene. a, Schematic of potential profile along the double-gated twisted bilayer devices. **b**, Electronic circuit model of the device. The applied gate voltages, V_t and V_b, drop across the electrolyte with capacitance C_t and C_b, yielding a potential V_{Gt} and V_{Gb} on the top and bottom layers, respectively. The two layers are capacitively coupled (C_m). **c**, Charge density in top layer as a function of gate voltages. Black curves, contour curves. Blue curve, n_t = 1x10^{14} cm^{-2} contour curve. **d**, Corresponding charge density map for bottom layer. **e**, Total charge density in the bilayer. Blue curve, n_{total} = 2x10^{14} cm^{-2}.

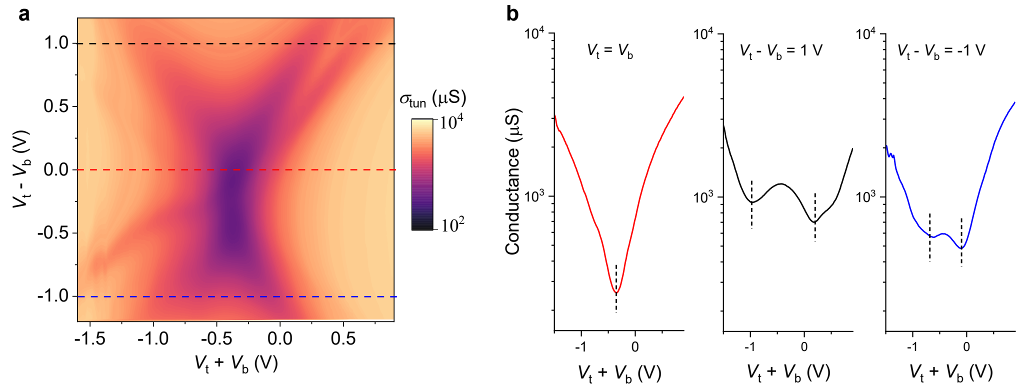

Supplementary Fig. 9 | Conductance map of twist bilayer graphene shows the splitting of the neutrality lines. a, Map of tunnelling conductance of twist bilayer graphene as a function $V_t + V_b$ and $V_t - V_b$. The black, red and blue dashed lines mark the cut for constant electric field with $V_t - V_b$ of 1 V, 0 and -1 V, respectively. **b,** the corresponding transfer curves showing the splitting Dirac point at 1 V or -1 V.
